# Elevated urinary levels of urokinase-type plasminogen activator receptor (uPAR) in pancreatic ductal adenocarcinoma identify a clinically high-risk group

**DOI:** 10.1186/1471-2407-11-448

**Published:** 2011-10-14

**Authors:** Claudio Sorio, Andrea Mafficini, Federico Furlan, Stefano Barbi, Antonio Bonora, Giorgio Brocco, Francesco Blasi, Giorgio Talamini, Claudio Bassi, Aldo Scarpa

**Affiliations:** 1 Department of Pathology and Diagnostics, University of Verona, Verona, Italy; 2 ARC-Net Research Center, University of Verona, Policlinico G.B. Rossi, Verona, Italy; 3 BoNetwork, San Raffaele Scientific Institute, Milan, Italy; 4 Department of Molecular Biology and Functional Genomics, San Raffaele Scientific Institute, Milan, Italy; 5 Department of Surgery, University of Verona, Verona, Italy; 6 Laboratory of chemical, clinical and haematological analyses, Hospitals of Verona, Verona, Italy

## Abstract

**Background:**

The urokinase plasminogen activator receptor is highly expressed and its gene is amplified in about 50% of pancreatic ductal adenocarcinomas; this last feature is associated with worse prognosis. It is unknown whether the level of its soluble form (suPAR) in urine may be a diagnostic-prognostic marker in these patients.

**Methods:**

The urinary level of suPAR was measured in 146 patients, 94 pancreatic ductal adenocarcinoma and 52 chronic pancreatitis. Urine from 104 healthy subjects with similar age and gender distribution served as controls. suPAR levels were normalized with creatinine levels (suPAR/creatinine, ng/mg) to remove urine dilution effect.

**Results:**

Urinary suPAR/creatinine values of pancreatic ductal adenocarcinoma patients were significantly higher (median 9.8; 25^th^-75^th ^percentiles 5.3-20.7) than those of either healthy donors (median 0; 0-0.5) or chronic pancreatitis patients (median 2.7; 0.9-4.7). The distribution of values among cancer patients was widespread and asymmetric, 53% subjects having values beyond the 95^th ^percentile of healthy donors. The values of suPAR/creatinine did not correlate with tumour stage, Ca19-9 or CEA levels. Higher values correlated with poor prognosis among non-resected patients at univariate analysis; multivariate Cox regression identified high urinary suPAR/creatinine as an independent predictor of poor survival among all cancer patients (odds ratio 2.10, p = 0.0023), together with tumour stage (stage III odds ratio 2.65, p = 0.0017; stage IV odds ratio 4.61, p < 0.0001) and female gender (odds ratio 1.85, p = 0.01).

**Conclusions:**

A high urinary suPAR/creatinine ratio represents a useful marker for the identification of a subset of patients with poorer outcome.

## Background

Pancreatic ductal adenocarcinoma (PDAC) is one of the leading causes of cancer-related death in the Western world [1,2]. Up to 80% of patients have locally advanced or metastatic disease at diagnosis; their median survival is 6 months and treatment, including chemotherapy and/or radiotherapy, have very limited benefit in terms of prolonging life [3,4]; the overall five year survival is less than 5% [2]. Patients who undergo surgery have a better prognosis and the addition of chemotherapy is more effective in extending their lives [5,6]. Earlier diagnosis can therefore have strong beneficial effect on survival.

Extensive research has focused on finding reliable diagnostic/prognostic molecular markers. Unfortunately, many of the candidate markers have been of no clinical use because of poor specificity and/or sensitivity [7], and the only FDA approved serum marker for pancreatic cancer to date remains CA19-9. New potential markers are being constantly tested and some have appeared to be superior to CA19-9, especially when used in combination [8-10]. The majority of these studies used plasma or serum. Urine, however, being a plasma ultrafiltrate, may contain cancer-derived molecules at a higher concentration than into the blood; it may also contain molecules that are quickly removed from the bloodstream and thus undetectable in plasma samples.

We focused on the uPA/uPAR system (urokinase-type plasminogen activator and its receptor). uPA is a serine protease that specifically activates plasminogen to plasmin. It is synthesized as an inactive precursor (pro-uPA) that undergoes proteolytic activation. Pro-uPA and uPA bind with high affinity to a specific receptor, uPAR (CD87), which is extracellularly docked to the plasma membrane by a glycosylphosphatidylinositol (GPI) anchor [11-13]. uPAR is constituted by three repeats (D1, D2 and D3), of about 90 residues each, connected by two linker regions and defining specific protein domains [14,15]. The linker region between domains D1 and D2 is highly susceptible to endoproteolytic cleavage by proteinases such as uPA itself, plasmin, elastase, matrix metalloproteinases and cathepsin G [16-19].

The binding of uPA to uPAR induces cell migration, adhesion and proliferation [13,20,21]. The soluble form of uPAR (suPAR), generated by proteases or cleavage of the GPI anchor by phospholipases, is essential for these processes: it behaves as a chemokine by binding either integrins or, in its cleaved form, a seven-transmembrane receptor (FPRL-1), attracting monocytes to the site of inflammation [13,22,23].

The uPA/uPAR system is also involved in cancer pathogenesis and soluble uPAR was first found in the blood and ascitic fluids of ovarian cancer patients; subsequently uPAR and its soluble form were respectively reported in tissues and in serum/plasma of patients with other cancer types [24-26]. Enhanced serum suPAR concentrations were indicative of poor prognosis in a group of ovarian carcinoma patients [27] and members of the plasminogen activator system including uPA, PAI-1 and uPAR itself, have been suggested to have prognostic value in a large number of human cancers [26,28]. SuPAR has been detected in urine of healthy women and patients with diverse ovarian-related diseases; in the same work the correlation between the plasma and urinary levels of suPAR was demonstrated. However, due to the limited number of samples analyzed, the diagnostic and prognostic value of these data was not assessable [29]. The same authors showed that primary tumour extracts contain both intact and cleaved suPAR but this last form is missing in serum samples from the same patients; it can however be detected, together with the intact molecule, in ascites and urine [30]. Urinary suPAR levels were also elevated in a group of patients affected by bladder carcinoma but differences were not distinct enough to reach significance as a diagnostic-prognostic marker [31].

The majority of pancreatic adenocarcinomas express urokinase-type plasminogen activator (uPA) and its receptor (uPAR) [32,33], but only about a half of them show a marked increase. This feature has been recently associated with uPAR gene amplification and defines a clinical group with poorer prognosis [34]. However, these studies only rely on pancreatic tissue specimens and the presence of suPAR has not been evaluated in urine of these patients. The purpose of this study was to investigate the presence of suPAR in urine of PDAC patients to define its diagnostic and prognostic significance.

## Methods

### Patients and healthy donors

According to published data, uPAR is markedly overexpressed only in a fraction of pancreatic cancer patients. We thus hypothesized that only a subgroup of patients would display increased urinary suPAR levels as well. To estimate the sample size we ran a preliminary power analysis; we hypothesized a difference in proportions of subjects with increased suPAR levels between patients and controls of 30%. A sample size of 100 patients and 100 controls was adequate by chi-square analysis with a power of 0.95.

We used plasma and urine from 94 PDAC patients (50 females, 44 males; mean age 60 yrs, SD 10), 52 chronic pancreatitis (15 females, 37 males; mean age 50 yrs, SD 12), and 104 healthy controls (48 females, 56 males; mean age 53 yrs, SD 19) enrolled from January 2004 to December 2006; all evaluable chronic pancreatitis cases were included. Written, informed consent was obtained according to a protocol approved by local ethics committee. Follow-up of patients ended in December 2009; PDAC patients evaluable for survival were 91. Median follow-up was 10.5 months (range 0.5-56.6) with 82 deaths of disease and 9 censored subjects exiting follow-up at different time points (1 dead of other causes, 2 alive with disease, 6 dropped out).

TNM stage (UICC/AJCC 2010) was available for 91 patients: stage I (T1,2 N0 M0: tumour limited to pancreas [T1,2], no regional lymph node metastasis [N0], no distant metastasis [M0]) n = 2, stage II (T3 N0 M0 or T1,2,3 N1 M0: tumour extends beyond pancreas [T3] and/or has lymph node metastases [N1] without involving the celiac axis or superior mesenteric artery) n = 21, stage III (T4 anyN M0: tumour involves the celiac axis or superior mesenteric artery [T4] but without distant metastasis) n = 34, stage IV (anyT anyN M1: tumour with distant metastasis [M1]) n = 34. Fifty patients (53%) underwent surgery, 21 of them were submitted to radical resections, 22 to palliative and 7 to explorative procedures. No patient had renal impairment according to serum creatinine measurements. Staging of inoperable patients was based on computed tomography, ultrasound imaging data and fine needle aspiration cytology. None of the enrolled patients had received chemotherapy before blood and urine collection. Ca 19-9 and CEA data were available in 79 of the 94 PDAC patients and their respective median values were 286.5 U/ml (range 1-58791) and 3.3 ng/ml (range 0.3-471.8). Performance status according to Karnofsky classification was available for 82 of 94 PDAC patients; its value was 100% for 14 patients, 80-90% for 58 patients and under 70% for 10 patients. The presence of cancer in chronic pancreatitis patients was excluded using imaging techniques (ultrasonography, CT scan, MR), endoscopy and fine needle aspiration cytology or biopsy. All patients were submitted to follow-up for a minimum of two years, according to a well-established procedure at our national referral centre for pancreatic disease[35,36].

### Collection and handling of samples

Blood and/or urine were collected from fasting patients upon hospitalization. Plasma was recovered after centrifugation of EDTA-treated blood for 5 min at 200 × g and further centrifuged for 30 min at 3000 × g to remove platelets and cellular debris. Urine was centrifuged for 30 min at 3000 × g. Samples were aliquoted in 1.5 ml tubes, snap-frozen in liquid nitrogen and stored at -80°C until they were analyzed. When used, aliquots were slowly thawed in ice, thoroughly mixed and centrifuged for 5 min at 16000 × g to remove precipitate if present. Measurements were done by personnel blinded to the patient diagnosis. Creatinine was measured with the CREA creatinine Jaffé method kit (Roche Diagnostics, Mannheim, Germany) and read on a Roche/Hitachi MODULAR ANALYTICS P800 according to the manufacturer guidelines. CA19-9 and CEA levels were determined upon patients hospitalization with the LIAISON^® ^CA19-9™ and CEA kits; both kits were used on a LIAISON^® ^reader (DiaSorin, Saluggia, VC Italy) according to the manufacturer instructions; these were standard diagnostic procedures of the Hospitals of Verona at the time of measurement.

### Capture ELISA assay for the detection of soluble uPAR

Soluble uPAR was detected in plasma and urine samples using the AssayMax Human Urokinase Receptor (uPAR) ELISA Kit (Assaypro, St. Charles, MO, USA), according to the manufacturer's guidelines. All Samples (plasma and urine) were diluted 1:5 in the supplied buffer and measured in duplicate. Absorbance was measured at 450 nm on a Synergy plate reader (Biotek, Winooski, VT, USA) and concentrations determined with the Gen5^® ^program (Biotek, Winooski, VT, USA).

The ability of the above kit to detect all isoforms of suPAR was confirmed by analyzing ten urine samples, with suPAR concentrations spanning the full range of detectable values, also with the method described by Resnati *et al *[37].

Briefly, black immunoassay plates (Maxisorp Nunc, Langenselbold, Germany) were coated for 16 h at 4°C with 100 µl/well of anti-uPAR monoclonal antibody R4 diluted 1 µg/ml, in 0.1M carbonate buffer, pH 9.5. After rinsing three times with 300 µl PBS containing 0.1% Tween^® ^20, the wells were treated for 30 min at 37°C with 100 µl 2% BSA in PBS and again washed three times with 300 µl PBS containing 0.1% Tween^® ^20. The wells were then incubated for 2 h at 37°C with 100 µl of either purified suPAR protein at different concentrations or 1:5 diluted samples in PBS, 1% BSA. After three washes with PBS/0.1% Tween^® ^20, the wells were incubated for 1 h at 37°C with 100 µl anti-uPAR polyclonal antibody SI369, 1 µg/ml in PBS/1% BSA. After three washes with PBS/0.1% Tween^® ^20, the wells were incubated for 1 h at 37°C with 100 µl goat anti-rabbit immunoglobulins/HRP conjugate (GE Healthcare, Milan, Italy). After three washes with PBS/0.1% Tween^® ^20, 100 µl of freshly made Amplex Red (Invitrogen, Milan, Italy) substrate solution (5 µM Amplex Red in 50 mM sodium phosphate, pH 7.4 with the addition of 10 µl H_2_O_2 _10 vol.) were added. After 1 hour incubation at room temperature in the dark, HRP activity was detected by measuring fluorescence with a microplate reader (Victor^3^, Perkin Elmer) set for excitation in the range of 530-560 nm and emission-detection at 590 nm. Each sample was corrected for background fluorescence. The antibody R4 was a kind gift by Dr. Gumilla Hoyer-Hansen [38]; the antibody SI369 was produced in our laboratory as previously described [37].

Comparison of the two methods by Pearson linear correlation yielded R^2 ^= 0.95 (p < 0.0001); mean difference between measurement was -0.3 ng/ml with a standard error of 1.2.

### Statistical and survival analysis

Global analysis of suPAR/creatinine values was performed using Kruskal-Wallis test followed by Mann-Whitney test for multiple comparisons among the patients groups. Statistical tests were corrected for the large number of ties in the healthy donors group. Proportions where analyzed by Fisher's exact test. Correlation between suPAR/creatinine and other parameters was analyzed using Spearman's rank correlation for ordinal or continuous variables.

For comparison of Kaplan-Meier survival curves we used Mantel-Cox log-rank test; for multivariate survival analysis we used stepwise Cox proportional hazards regression; selection of the best model was performed using the "backward elimination" algorithm. For all the analyses a p-value below 0.05 was considered as significant. Graphs and univariate analyses were performed using Graphpad Prism^® ^version 5.00 for Mac (GraphPad Software, San Diego California USA, http://www.graphpad.com) and Gnumeric software (http://projects.gnome.org/gnumeric), multivariate Cox regression was done with R (version 2.10) using survival library (version 2.35.8) [39,40].

## Results

### Plasmatic and urinary suPAR levels are correlated

**Figure 1:**
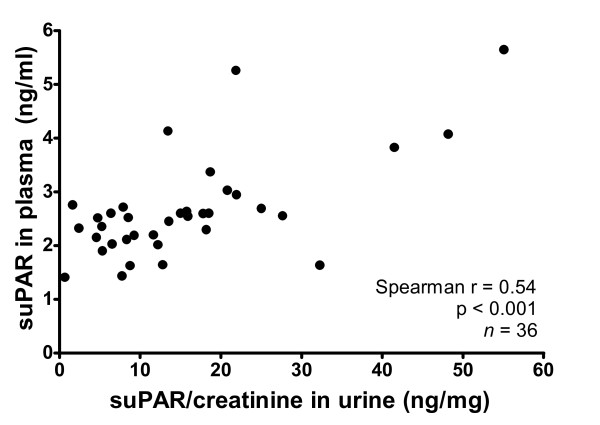
**Correlation between plasma suPAR concentration and urinary suPAR/creatinine level in pancreatic adenocarcinoma patients**.

The correlation of plasmatic and urinary suPAR values (Figure 1) was assessed using the first 36 PDAC patients. A significant though limited correlation between the two parameters was found (Spearman r = 0.54; p < 0.001; n = 36), thus showing that urinary suPAR levels vary in accordance with plasmatic levels only to a limited extent. Moreover, 13 of 21 patients showed elevated urinary suPAR without a corresponding increase in plasmatic levels. Given this difference and since urines are the only of the two sample types where both intact and cleaved suPAR accumulate according to published data [30], we measured only urinary suPAR for the remaining patients. The plasmatic levels of suPAR are herein reported in ng/ml, while the levels of urinary suPAR levels are reported as suPAR/creatinine ratio and thus expressed as ng/mg. We used creatinine normalization, a standard clinical practice for many urinary markers, to correct the effect of differences in dilution of the urine (due to water reabsorption) and to improve the consistency of the assay, since urinary excretion of any biomarker is affected by the glomerular filtration rate and the resultant urinary flow. This method has been described for urinary suPAR by Sier *et al *[29].

### Urinary suPAR levels in PDAC patients are higher than in CP patients and healthy controls

We measured the levels of suPAR/creatinine in the urine of 146 patients affected by pancreatic diseases and 104 healthy donors. The 146 patients included 94 affected from pancreatic ductal adenocarcinoma and 52 from chronic pancreatitis. Clinical data of PDAC patients are summarized in Table 1.

**Table 1:** Clinical data of pancreatic adenocarcinoma patients

Characteristics	Value
Number of patients	94
Males/Females	44/50
Mean Age (SD), years	60 (10)
Subjects with follow-up data available	91
Censored subjects (Drop-outs)	9 (6)

**Surgery**	**n**
Resected	21
Not resected	
Palliative surgery	22
Explorative surgery	7
Not operated	41

**Stage**	**n**
I (T1,2 N0 M0)	2
II (T3 N0 M0 or T1,2,3 N1 M0)	21
III (T4 anyN M0)	34
IVB (anyT anyN M1)	34

**Follow-up from diagnosis, median (25^th^-75^th ^percentiles)**	**Months**
Overall	10.5 (5.3-18.5)
Resected (n = 21)	22.7 (13.7-31.8)
Not resected (n = 70)	7.9 (5.0-15.5)

**Figure 2:**
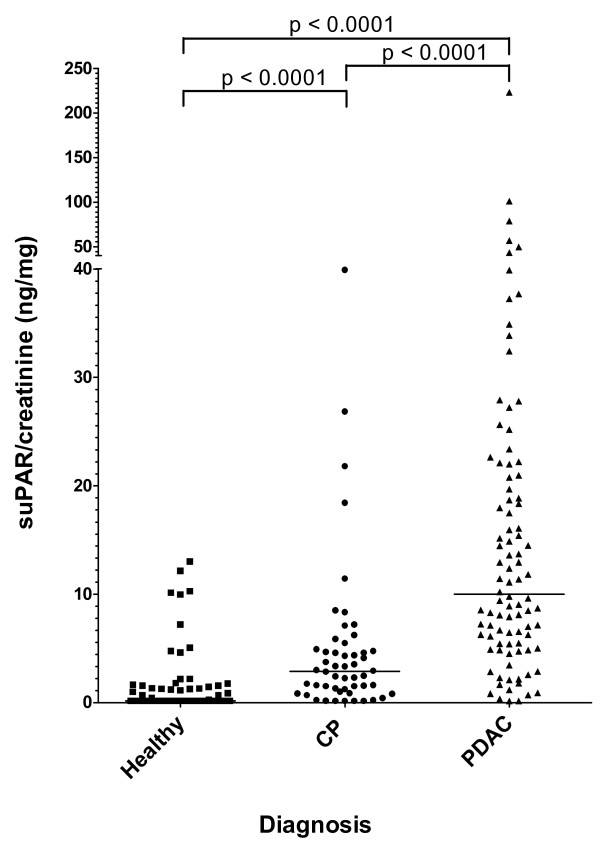
**Urinary suPAR/creatinine levels in patients and controls**. Scatter plot showing urinary suPAR/creatinine levels in: Healthy = healthy donors, CP = chronic pancreatitis, PDAC = pancreatic ductal adenocarcinoma. Bars indicate median values. Post-hoc comparisons (Mann-Whitney test) between clinical groups are indicated in the upper bars.

A scatter plot of the values of suPAR/creatinine in the two groups of patients and in healthy donors is shown in Figure 2, while a summary of suPAR data for patients and controls is presented in Table 2. Healthy controls bore low or undetectable levels of suPAR/creatinine (median 0; 25^th^-75^th ^percentiles 0-0.5), with 95% of the subjects below 9.1 ng/mg. The values of PDAC (median 9.8; 5.3-20.7) and CP (median 2.7; 0.9-4.7) groups were compared to each other and to those of healthy controls by Kruskal-Wallis test followed by Mann-Whitney post-hoc test and resulted to be significantly (p < 0.0001) different. The distributions of values measured in PDAC patients, CP patients and healthy controls were asymmetric, with skewness of 5.48, 3.40 and 3.25 respectively; a fraction of PDAC patients displayed high suPAR/creatinine levels while the rest had values close to CP patients and healthy donors. To better distinguish PDAC patients with increased suPAR/creatinine levels, considering the ROC curve analysis, we took the 95^th ^percentile of healthy donors as a threshold and calculated the proportion of subjects with either high (> 9.1 ng/mg) or low (≤ 9.1 ng/mg) suPAR/creatinine ratio in each group. This threshold was used for all the subsequent analyses as a mean to distinguish patients with abnormally increased suPAR/creatinine. As Table 2 shows, 53.2% of PDAC patients had values of suPAR/creatinine higher than the threshold while only 9.6% of CP patients had values beyond the threshold; the proportions of subjects with high values of suPAR/creatinine were compared to healthy donors by Fisher's exact test and only PDAC patients showed a significant difference (p < 0.0001).

**Table 2:** Summary of suPAR/creatinine data for patients and controls

	Healthy Controls	Chronic Pancreatitis	Ductal Carcinoma
Number of cases	104	52	94
Mean Age (SD)	53 (19)	50 (12)	60 (10)
Males/females	56/48	37/15	44/50
suPAR/creatinine median (25^th^-75^th ^percentile)	0 (0-0.5)	2.7 (0.9-4.7)	9.8 (5.3-20.7)
Comparison with controls median*	-	p < 0.0001	p < 0.0001
Elevated suPAR/creatinine°	4.8%	9.6% (p = 0.32)	53.2% (p < 0.0001)

°Value beyond 9.1 ng/mg (healthy donors' 95^th ^percentile) was used as a threshold, comparison with proportions of healthy controls by fisher's exact test.

* Mann-Whitney test

Finally, comparing proportions of subjects with high urinary suPAR/creatinine levels in healthy individuals and CP patients vs. PDAC patients, the specificity of the test was 93.6% (95% CI 88.6-96.5), with a sensitivity of 53.2 (95% CI 43.2-62.9).

### Urinary suPAR/creatinine ratio shows no correlation with tumour stage, CA19-9 and CEA

We verified the correlation between urinary suPAR/creatinine levels and other commonly used diagnostic parameters for PDAC patients. Spearman analysis showed no correlation between values of suPAR/creatinine and either CA19-9 (r = 0.195; p = 0.082) or CEA (r = 0.103; p = 0.337). Similarly, there was no significant association between suPAR/creatinine values and age (Spearman r = -0.117; p = 0.489). As for categorical variables, we performed the analysis by splitting patients with high (> 9.1 ng/mg) or low (≤ 9.1 ng/mg) suPAR/creatinine ratio, according to the above chosen threshold. The proportions of patients with elevated suPAR/creatinine were not significantly different in the subgroups of resectable (stage I/II), locally advanced (stage III) and metastatic (stage IV) subjects. Indeed, patients with high urinary suPAR/creatinine were 14 of 23 (61%) among stageI/II, 20 of 34 (59%) among stage III and 13 of 34 (38%) among stage IV; the group of stage IV patients displayed a smaller proportion of subjects with high suPAR/creatinine levels, although this difference was not statistically significant (Fisher's exact test; p = 0.17). As for gender, 25 of 43 male (58%) patients and 22 of 48 (46%) female patients showed high suPAR/creatinine levels (Fisher's exact test; p = 0.29). Similarly, no association was found between high suPAR/creatinine and performance status of patients: six of ten (60%) patients with a performance status under 70% had elevated urinary suPAR/creatinine compared to 28 of 58 (48%) patients with a performance status of 80-90% and 10 of 14 (71%) patients with a performance status of 100% (Fisher's exact test; p = 0.28).

### Increased urinary suPAR/creatinine ratio is an independent adverse prognostic factor among non-resected pancreatic cancer patients

To assess the prognostic significance of high urinary suPAR/creatinine, PDAC patients' data were analyzed by multivariate stepwise Cox regression. Variables tested as independent predictors of survival were a high (> 9.1 ng/mg) urinary suPAR/creatinine level, tumour stage, gender and age. Tumour stage was strongly linked to resection, since all 23 stage I or II patients but two had been resected while all 68 stage III or IV patients had not; therefore differences between stages I-II and III-IV also included resection's effects. The results are summarized in Table 3: an elevated urinary suPAR/creatinine level resulted to be associated with poorer survival with an odds ratio of 2.10 (p = 0.0023). Other variables that significantly affected overall survival were female gender (odds ratio 1.85) and tumour stage, the latter showing the most relevant prognostic impact (stage III odds ratio 2.65; stage IV odds ratio 4.61). Both stages III and IV showed worse survival compared to lower stages (i.e. patients that had been resected); however, confidence intervals of odds ratios for stages III and IV overlapped, indicating that differences in survival between these two groups of patients didn't reach statistical significance.

**Table 3:** Results of Cox regression analysis of pancreatic ductal adenocarcinoma patients (n = 91)

Variable	Odds Ratio	95% CI	p
Gender = female	1.85	1.16 - 2.96	0.01
Stage = I, II	1	-	-
Stage = III	2.65	1.45 - 4.87	0.0017
Stage = IV	4.61	2.42 - 8.76	< 0.0001
High (> 9.1 ng/mg) suPAR/creatinine	2.10	1.31 - 3.36	0.0023

Age	1.02	1.00-1.04	0.067

**Figure 3:**
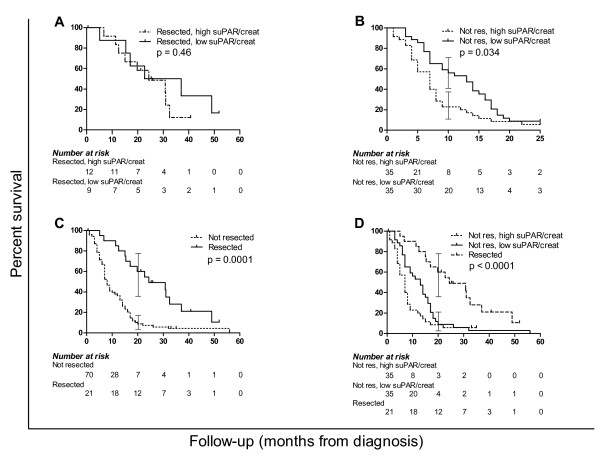
**Univariate survival analysis of pancreatic adenocarcinoma patients**. Kaplan-meier analysis of pancreatic adenocarcinoma patients based on surgery and suPAR/creatinine levels; curves compared by Mantel-Cox log-rank test, brackets illustrate 95% CI of survival. A) survival difference between resected patients bearing levels of suPAR/creatinine higher or lower than 9.1 ng/mg (95^th ^percentile of healthy donors) is not significant (p = 0.46). B) Difference in survival between non resected patients according to suPAR/creatinine levels (p = 0.034). C) Survival difference between resected and non-resected patients without considering suPAR/creatinine levels (p = 0.0001). D) Survival of resected vs. non-resected patients with either low or high suPAR/creatinine (log-rank test p < 0.0001, log-rank test for trend between curves p = 0.014).

At univariate Kaplan-Meier survival analysis, patients with a high urinary suPAR/creatinine level showed a trend to poorer survival (median survival of 8 months vs. 14 months of patients with low suPAR/creatinine). However, the two survival curves overlapped after 18 months yielding a not significant (p = 0.20) log-rank test. Considering the strong impact of resection on overall survival and tumour progression, the analysis was also performed after stratifying patients by resection as shown in Figure 3. The small subgroup of resected patients (n = 21) had no significant difference in survival according to suPAR urinary levels, while non-resected patients survival curves showed a significant separation (p = 0.034). After further stratification of unresectable patients into metastatic (n = 34) vs. not metastatic (n = 36), patients with high suPAR/creatinine levels still showed a trend to poorer survival in both subgroups. Indeed, metastatic patients with high suPAR/creatinine had a median survival of 7 months vs. 14 months of the ones with low suPAR/creatinine; not metastatic patients with high suPAR/creatinine had a median survival of 7 months vs. 12 months of those with low suPAR/creatinine. However, the survival differences only approached statistical significance (log-rank test: p = 0.08 for metastatic patients; p = 0.1 for not metastatic patients). Taken together, curves of resected and non-resected patients with either low or high suPAR/creatinine (Figure 3D) were significantly separated (p < 0.0001), showing a trend to progressively worse prognosis (p = 0.014). Median survivals were respectively 24 months from diagnosis for resected patients, 13 months for non-resected with normal suPAR/creatinine, 7 months for non-resected with high suPAR/creatinine.

## Discussion

We report in the present study that high levels of suPAR in the urine of patients suffering from pancreatic ductal adenocarcinoma identify a clinically high risk subgroup.

Indeed, 53% of our 94 patients had urinary suPAR/creatinine values above the 95^th ^percentile of healthy donors at 9.1 ng/mg, while the remaining 47% had suPAR/creatinine levels below that value. This threshold for urinary suPAR/creatinine was capable of identifying a group of patients with worse prognosis. Unresected patients displaying high urinary values of suPAR/creatinine showed a significantly increased mortality at univariate survival analysis, with surviving patients dropping to 22% after 9 months; at the same time point, 60% of the patients with low suPAR urinary levels were still alive. The association between high urinary suPAR/creatinine levels and poorer survival was not evident among resected patients at univariate analysis, possibly due to the small sample size (n = 21) of this patients group. However, the link between high urinary suPAR levels and worse prognosis was confirmed by multivariate survival analysis: Cox regression including both resected and unresected patients identified a high suPAR/creatinine level as an independent predictor of poor prognosis (odds ratio = 2.10).

The indication by multivariate survival analysis that urinary suPAR/creatinine gives additional prognostic information independent of that given by tumour stage is further supported by proportions analysis that showed the absence of correlation between tumour stage and suPAR/creatinine. In fact, patients with resectable (stage I and II), locally advanced (stage III) or metastatic (stage IV) tumours showed a similar proportion of cases displaying high suPAR/creatinine urinary levels (p = 0.17).

Our work showed that half of PDAC patients had markedly elevated suPAR/creatinine values and poorer prognosis, while the other half of PDAC patients had values comparable to those of chronic pancreatitis patients and healthy subjects. Interestingly, previous immunohistochemical studies reported that the level of uPAR in primary pancreatic adenocarcinoma was higher than in normal pancreas and tended to be associated with a worse prognosis [32-34]. In particular, Hildenbrand *et al *showed that virtually all (48 of 50) primary carcinomas overexpress uPAR compared to normal pancreatic tissue by immunohistochemistry, and that 52% of them had uPAR gene amplification by FISH analysis. This latter feature correlated with worse prognosis and with a higher protein concentration in the tumour as quantitatively measured by ELISA on tissue lysates; conversely, tumours without gene amplification and chronic pancreatitis samples had comparably lower concentrations of uPAR [34].

Compared to its measurement on the tumour tissue, the measurement of suPAR in urine provides at least three advantages. First, the measurement does not require tumour biopsies, and can thus be performed on both resectable subjects, before and after surgery, and on unresected patients. Second, the measurements can be taken repeatedly and may be used in the follow-up of patients. Third, the samples are obtained by absolutely non-invasive methods, reducing the need for patient hospitalization. One drawback of urinary markers is their dependence on glomerular filtration rate: in the case of renal impairment or failure, the ratio between creatinine and protein markers levels become unreliable.

Our data also show that suPAR/creatinine values are independent from other commonly used diagnostic and prognostic parameters (CA19-9, CEA), and thus can be used in synergy with them, providing an additional layer of information. As for its diagnostic performance, a high (>9.1 ng/mg) urinary suPAR/creatinine ratio showed 53.2% sensitivity and 93.6% specificity in diagnosing pancreatic adenocarcinoma. Given its high positive predictive value and considering a doubtful clinical situation in which other parameters do not yield a clear answer, a high urinary suPAR/creatinine level may be useful in the decision making process.

## Conclusions

Our data show that suPAR urinary levels are increased in a large subgroup of patients suffering from pancreatic ductal adenocarcinoma and such an increase is rarely seen among patients with chronic pancreatitis. This increase is associated to poorer survival and the prognostic information given by urinary suPAR levels is independent of that furnished by tumour staging. Thus, a high urinary suPAR/creatinine ratio represents a useful marker for the identification of a subset of patients with clinical high risk of poorer outcome.

## Abbreviations

PDAC: pancreatic ductal adenocarcinoma; CP: chronic pancreatitis; uPA: urokinase plasminogen activator; uPAR: urokinase plasminogen activator receptor; suPAR: soluble urokinase plasminogen activator receptor.

## Competing interests

The authors declare that they have no competing interests.

## Authors' contributions

CS and AM contributed to experimental design, data collection, analysis and drafted the manuscript. FF contributed to analysis. SB and GT contributed to data analysis and revised the manuscript. AB and GB contributed to sample and data collection, FB and CB contributed to data interpretation, AS coordinated the study and finalized the manuscript. All authors read and approved the final manuscript.

## Pre-publication history

The pre-publication history for this paper can be accessed here:

http://www.biomedcentral.com/1471-2407/11/448/prepub
